# The unique contributions of Rab11 and Rab35 to the completion of cell division

**DOI:** 10.1186/s40659-025-00638-x

**Published:** 2025-08-29

**Authors:** Paulius Gibieža, Emilija Ratkevičiūtė, Girstautė Dabkevičiūtė, Vilma Petrikaitė

**Affiliations:** Laboratory of Drug Targets Histopathology, Institute of Cardiology, Lithuanian University of Health Sciences, Sukilėlių Av. 13, 50162 Kaunas, Lithuania

**Keywords:** Rab11, Rab35, Division, Abscission, Telophase, Cytokinesis

## Abstract

Rab11 and Rab35 have been implicated in large-scale intracellular membrane trafficking during the last phases of the cell cycle. Although both proteins are associated with cytokinetic abscission, they appear to perform distinct functions and give rise to different phenotypes in dividing cells. Despite a substantial body of research on each protein individually, no study to date has systematically compared Rab11 and Rab35 in the context of cancer cell division. As a result, the extent of their interrelationship and potential compensatory mechanisms remains unclear. Our data demonstrate that Rab11a, Rab11b and Rab35 expression levels are partially interrelated. We also show that Rab11 and Rab35 contribute to mitotic progression in different ways, particularly during specific stages of the M-phase. Notably, depletion of either Rab11 or Rab35 disrupts cytokinetic abscission and correlates with aberrant F-actin accumulation at the intercellular bridge. Furthermore, overexpression of related Rab proteins with overlapping functions does not rescue the cytokinetic defects caused by Rab11 or Rab35 downregulation in cancer cells. Therefore, this study aims to deepen our understanding of how Rab11 and Rab35 orchestrate the molecular events that drive the progression from late anaphase through the completion of cytokinesis.

**Supplementary Information:**

The online version contains supplementary material available at 10.1186/s40659-025-00638-x.

## Background

Cytokinesis occurs at the end of mitosis, when the cytoplasm that connects two newly formed daughter cells is cleaved in two, separating the cells [1–3]. For the successful division to occur, cells must experience a tremendous number of cytoskeletal modifications and highly coordinated intracellular processes, which would allow for smooth and equivalent distribution of cellular compartments between the two, resulting in a physical separation of dividing cells. The actual cessation of the newly forming cells starts in late anaphase when the cell cytoplasm undergoes contraction via the formation of an actomyosin ring made of filamentous actin (F-actin), motor protein myosin-II, formins and many actin cross-linkers [4–6]. The actomyosin-based contractile ring forms under the plasma membrane's surface and is positioned so that it can separate chromosomes. However, the constriction of the plasma membrane alone is insufficient to separate newly formed cells. When the contractile ring constricts to its absolute capacity, it forms an intercellular bridge (ICB), which now possesses large amounts of F-actin and a mass of highly compacted central spindle microtubule bundles.

For some time, multiple research groups have been trying to clarify the regulation of molecular machinery that drives the removal of actin deposits and central spindle microtubules from the ICB during cytokinesis. Here, Rab GTPases were shown to deliver specialised membranous vesicles and various proteins mediating cytokinesis [7–9]. The considerable attention that this topic has drawn emphasises the importance of this process to cell life and homeostasis as a unit of the whole system, which, if misregulated, can lead to various diseases, including cancer [10–12]. In particular, this is relevant to the Rab GTPases-mediated cytokinesis event, which, upon dysregulation, can cause aneuploidy and multinucleation, eventually inducing tumorigenesis [13–15]. In principle, successful cytokinesis requires spatial and temporal membrane trafficking, which Rab11 and Rab35 primarily regulate from the entire Rab protein family. These molecular switches belong to a family of small monomeric GTPases, so they cycle between active-GTP-bound and inactive-GDP states. When GTP-bound via its effectors FIP3 and FIP4, Rab11 targets intracellular vesicles derived from recycling endosomes along the central spindle microtubules to the cleavage furrow during late cytokinesis [16, 17]. Normally, Rab11-containing endosomes are distributed throughout the cytoplasm during metaphase and anaphase. As the mitotic phase progresses towards the end, these Rab11 endosomes are concentrated at the cleavage furrow and regulate telophase and final cytokinetic division in mammalian cells [16]. This mediates cortical actin depolymerisation at the abscission site by recruiting and transporting SCAMP2/3 and p50RhoGAP to the ICB. As a result, it inhibits RhoGTPase activity and reduces F-actin polymerisation, narrowing the ICB [18, 19]. Additionally, Rab11-FIP3 and FIP4 complexes were shown to interact with Arf6 and regulate membrane trafficking to the sites of the ICB and the midbody (MB) before the final abscission [17, 20].

Rab35 also shares Rab11’s function in facilitating F-actin removal from the abscission site. Rab35-containing endosomes are found throughout the cytoplasm during interphase and accumulate at the ingressing furrow and ICB during early and late cytokinesis, respectively [21]. It lowers the amount of phosphatidylinositol-4,5-bisphosphate (PtdIns(4,5)P_2_), which otherwise would maintain the stability of the existing ICB via the promotion of actin polymerisation [22]. To stop this process, Rab35 recruits its effector, Oculo-Cerebro-Renal syndrome of Lowe (OCRL) lipid phosphatase, which targets OCRL to the ICB, where local hydrolysis of PtdIns(4,5)P_2_ finally occurs. In this way, and because of the reduced cortical F-actin oligomerisation, the F-actin levels at the ICB are kept at a level sufficient for normal cytokinetic abscission to occur [21, 22]. Furthermore, Rab35 recruits another effector, MICAL1 oxidoreductase, which catalyses the oxidation of the two methionine residues of F-actin (M44 and M47). It thus activates the depolymerisation of actin filaments at the abscission site [23–25], which narrows the ICB to such an extent that it is required for the incoming microtubule-severing enzyme spastin to bind to CHMP1B. Then, this molecular interaction activates the ESCRT-III machinery, targeted to the MB, to coordinate the mitotic spindle disassembly [26–28]. CHMP4B assembles with the ESCRT-III subunit at the end of cytokinetic abscission and forms the cortical membrane-distorting helical filaments. This process mediates the complete termination of the remaining ICB at the secondary ingression site. It finally separates newly formed daughter cells [29–32].

Rab11 and Rab35 also possess a wide range of roles outside of cytokinesis regulation. These roles include regulating cell migration, neurite outgrowth, morphogenesis, apico-basal polarity, immune synapse modulation, phagocytosis, endocytosis, etc. To give a more detailed example, the Rab11 family member Rab25 was shown to mediate the formation of F-actin-rich filopodia-like protrusions to promote cancer cell invasive migration in the ovarian cancer cell line A2780 [33]. Exploring further roles, Rab11 was shown to regulate vesicle trafficking required to form ducts with continuous lumens during organ morphogenesis, particularly during pancreatic development [34]. Another function revealed by *Zhu* et al., where Rab11 was demonstrated to be involved in the formation of tunnelling nanotube structures between Schwann cells that affect peripheral nerve regeneration via the regulation of neural cell connections [35]. Like Rab11, Rab35 is also implicated in cell migration; it promotes migrasome formation on the ends of retraction fibres at the trailing edge of migrating cells by recruiting and accumulating integrin α5 at the migrasome formation sites [36]. Moreover, Rab35 was shown to play a role in polarity initiation and apical lumen positioning during the first cell division of cyst development [37]. Inactivation of Rab35 results in a complete inversion of apico-basal polarity in 3D cysts [37]. As a final example, Rab35 was shown to colocalise with F-actin and Rho-family GTPase Rac1, which indicates that Rab35 is involved in neurite outgrowth by regulating Rho-family GTPase activity [38].

Despite all the data available, the literature lacks information about the specific phenotypic differences that Rab11 and Rab35 confer to dividing cancer cells. Also, nobody looked at the possible interrelationship between these two proteins during the regulation of processes necessary for cells, especially during cell division. The only attempt to demonstrate the interaction between Rab11 and Rab35 was by *Iannantuono* et al., where Rab11-FIP1 was shown to maintain Rab35 at the ICB to mediate actin removal during abscission. Respectively, the absence of Rab11-FIP1 was shown to reduce Rab35 and further cytokinetic defects [39]. Since Rab11 and Rab35 play functionally similar roles during cell division [18, 40], it would be interesting to see whether the individual or simultaneous downregulation of both, in terms of the regulation of cytokinesis, would have any effect on the progression of cancer cell division. Another intriguing point would be determining whether downregulation-associated phenotypic defects in cancer cells could be rescued by overexpressing the same or functionally similar gene.

For the above reasons, we used an RNAi approach and vector-based short hairpin RNA (shRNA) interference to downregulate an individual Rabs and different combinations of Rab11a, Rab11b, and Rab35. We also carried out localisation studies, functional assays, and rescue experiments to characterise the division-stage phenotype of both control and mutated cervical cancer cells.

## Methods

### Cell culture

The human adenocarcinoma HeLa-wt cell line was obtained from the American Type Culture Collection (ATCC, Manassas, USA). HeLa-wt cells were maintained in 5% CO_2_ and at 37 °C in Dulbecco's modified Eagle's medium (DMEM, Life Technologies Gibco, USA), supplemented with 10% fetal bovine serum (Life Technologies Gibco, USA) and a 1% solution of 10,000 units/ml penicillin and 10 mg/ml streptomycin (SIGMA, USA). Cells were regularly tested for Mycoplasma contamination.

### Generation of lentiviral stable cell lines

Lentiviral transduction particles were acquired from Sigma Aldrich (USA). Target sequences used for shRNA treatment are as follows: Non-targeting shRNA control (#SHC016V-1EA), Rab11a—5’-GCCTTATTGGTTTATGACATT-3'(#TRCN0000073022), Rab11b—5’-GCCTTGGATTCCACTAACGTA-3'(#TRCN0000029184), and Rab35—5’-AGAGCAGTTTACTGTTGCGTT-3'(#TRCN0000047796). Different lentiviral particles mixed with media were pre-treated with polybrene (Merck Millipore, USA, #TR-1003-G) before being applied overnight to 50% confluent target HeLa-wt cells. Cells were allowed to recover for 24 h, then selected with puromycin (3 µg/ml), expanded and frozen as stocks. Low-passage cells were used in all experiments to ensure experimental consistency. The remaining expression of Rab genes of interest in cell lines was then validated using Western Blotting.

### siRNA treatments

HeLa-wt cells were transfected with various siRNAs using Lipofectamine 2000 (Invitrogen, USA, #11668019). The double-stranded RNA sequences used for siRNA treatment are as follows: Silencer Select Negative Control #1 siRNA (Ambion, USA, #4390843), Rab11a—5’-CAACAAUGUGGUUCCUAUUtt-3'(Ambion, USA #4390824), Rab11b—5’-CUAACGUAGAGGAAGCAUUtt-3'(Ambion, USA #4390824), Rab35—5’-GCAGUUUACUGUUGCGUUUtt-3'(Ambion, USA #4390824). Transfection of individual siRNAs, combinations of different siRNAs, and co-transfection of plasmid DNA with siRNA for rescue experiments were performed following the manufacturer’s (Thermo Fisher Scientific, USA) guidelines. At least three independent biological replicates were performed for each siRNA transfection. Data are reported as the mean with the corresponding standard deviation.

### Rescue assay

To rescue the phenotypic variations associated with Rab11a and Rab35 downregulation in HeLa-wt cells, siRNA-treated cells plated on collagen I-coated (at a final 50 µg/ml concentration, Life Technologies Gibco, USA) No. 1 microscope cover glasses (VWR) were transfected with either siRNA-resistant Rab11a-GFP (pEGFP-C1-Rab11a) or Rab35-GFP (pEGFP-C1-Rab35) plasmids using Lipofectamine 2000 (Invitrogen, USA, #11668019). Transfections were performed according to the manufacturer's protocol.

The pEGFP-C1-Rab11a wt and pEGFP-C1-Rab35 wt plasmids used in the rescue experiments were kindly provided by Prof. Rytis Prekeris, University of Colorado Anschutz Medical Campus, Denver, USA.

### Western blotting

For Western blotting, cells were scraped in 1 × PBS containing 1 mM PMSF (Thermo Fisher Scientific, USA, #36978) and 1% Triton X-100 (Thermo Fisher Scientific, USA, #A160046.AE) and incubated for 30 min. on ice. The collected cells were centrifuged at 15,000 g for 5 min. at 4 °C to pellet the lysates. The total protein was determined using a Bradford assay reagent (Thermo Fisher Scientific, Pierce, USA, #23238). Samples were mixed with 5 × SDS sample loading buffer (Thermo Fisher Scientific, Pierce, USA, #39000) and boiled for 5 min. at 95 °C. Depending on their molecular weight, the boiled samples were loaded onto an SDS-PAGE gel (12%) to separate proteins. Proteins were then transferred onto PVDF membranes and stained with the indicated antibodies under standard laboratory procedures. The expression of each protein was normalised to β-tubulin, used as the internal loading control. Protein quantification was performed through densitometric analysis with ImageJ software (https://imagej.nih.gov/ij/). Unless otherwise indicated, all data are derived from at least three independent experiments (biological replicates), calculating the mean and standard deviation.

### Immunofluorescence staining

For immunofluorescence analysis, cells plated on collagen I-coated (at final 50 µg/ml concentration, Life Technologies Gibco, USA) No. 1 microscope cover glasses (VWR) were fixed using 4% Paraformaldehyde (PFA) for 15 min. at room temperature (R.T.). Cells were then quenched for 5 min. with a Quench buffer (375 mg of glycine diluted in 1 × PBS), permeabilised and blocked in the incubation buffer (1% BSA, 1% Saponin, 2% FBS, all mixed in 1 × PBS) for 30 min., at R.T. Next, the cover glasses were incubated with primary antibodies for 1 h at 37 °C, in a moisture-maintaining incubator. Then, the cover glasses were incubated with secondary antibodies for 30 min. at 37 °C, in a moisture-maintained incubator. Finally, the cover glasses were incubated with 1 µg/ml DAPI (Thermo Fisher Scientific, USA, #D1306) solution for 5 min., at R.T. Lastly, the cover glasses were mounted on microscopic slides with a Prolong Diamond Antifade mounting media (Invitrogen, USA, #P36965).

Immunofluorescence was detected using an inverted fluorescence Olympus IX73 microscope (Olympus Europe Holding Gmbh) using a 20 × lens for multinucleation and mitotic stage analysis assays, and upright confocal Olympus Fluoview FV1000 microscope (Olympus Europe Holding Gmbh) using either a 20 ×, 40 × and 60 × oil lens for actin fluorescence intensity measurements and localisation assays, depending on the requirements. Three biological repeats were completed for each data set, each biological repeat containing 5 to 10 technical replicates. Data are reported as the mean with the corresponding standard deviation.

### Antibodies and staining reagents

The following primary antibodies were used for immunofluorescence and Western blotting: acetylated-α-tubulin (Cell Signalling, USA, #D20G3, IF-1:200), anti-Rab11a (Sigma Life Science, USA, #HPA051697, WB-1:250), anti-Rab11a (Nordic Biosite, Sweden, #ABB-FPO8IL-10, IF-1:250), anti-Rab11b (Sigma Life Science, USA, #HPA054396, WB-1:250), anti-Rab35 (Abcam, USA, #ab300116, WB-1:250), anti-Rab35 (Abcam, USA, #ab152138, IF-1:250), beta-tubulin loading control (Invitrogen Thermo Fisher Scientific, USA, #MA5-16308, WB-1:2000).

The following secondary antibodies were used for immunofluorescence: Alexa Fluor 488 donkey anti-rabbit IgG (H + L) (Thermo Fisher Scientific, USA, #A21206, IF-1:250), Alexa Fluor 488 goat anti-mouse IgG (H + L) (Thermo Fisher Scientific, USA, #A-11001, IF-1:200), Alexa Fluor 568 goat anti-rabbit IgG (H + L) (Abcam, USA, #ab175471, IF-1:200).

The staining dyes used for immunofluorescence include Alexa Fluor phalloidin 568 (Thermo Fisher Scientific, USA, #A12380, IF-1:50 from a 40 × stock) and DAPI (Thermo Fisher Scientific, USA, #D1306, IF-1 µg/ml).

### Multinucleation assay

To study multinucleation in siRNA-treated cells, HeLa-wt cells transfected with non-targeting-siRNA control were seeded on collagen I-coated (at final 50 μg/ml concentration, Life Technologies Gibco, USA) No. 1 microscope cover glasses (VWR, USA), which, after 72 h in culture, were fixed in 4% PFA (Thermo Fisher Scientific, USA). For various siRNA-treated cells, 24 h post-seeding on collagen I-coated glass coverslips, HeLa-wt cells were treated with appropriate siRNA constructs. This allowed for 48 h for siRNA expression, followed by fixation as previously described. Fixed cells were permeabilised with 0.2% Triton X-100 (Thermo Fisher Scientific, USA, #A160046.AE) for 3 min. at R.T. and stained with Alexa Fluor 568 phalloidin (Thermo Fisher Scientific, USA, #A12380) for 30 min. at 37 °C and with 1 µg/ml DAPI (Thermo Fisher Scientific, USA, #D1306) for 5 min. at R.T. The cover glasses were mounted on glass microscope slides using Prolong Diamond Antifade mountant (Invitrogen, USA, #P36965). Then, random fields on the coverslips were photographed using an inverted fluorescence Olympus IX73 microscope (Olympus Europe Holding GmbH) with a 20 × lens. The number of multinucleated cells, including binucleated, poly-lobed, and micronuclei, was counted manually. The rate of total multinucleated cells was then calculated in ten randomly chosen fields for each biological repeat. All cells were valued as one population. All data are derived from at least three independent experiments (biological repeats), each containing 5–15 technical replicates. Data are reported as the mean with the corresponding standard deviation.

To rescue multinucleation, different siRNA constructs together with respective GFP-tagged Rab11a- or Rab35-coding plasmids were transfected into HeLa-wt cells seeded on collagen I-coated No. 1 microscope cover glasses (VWR, USA) according to Lipofectamine 2000 manufacturer's (Invitrogen, USA, #11668019) instructions. Then, 48 h post-transfection, cells were processed and examined for multinucleation as described above. All data are derived from at least three independent experiments (biological repeats), each containing 5–15 technical replicates. Data are reported as the mean with the corresponding standard deviation.

To study multinucleation in shRNA-treated cells, stable cells individually depleted for Rab11a, Rab11b or Rab35 were seeded on collagen I-coated (at final 50 μg/ml concentration, Life Technologies Gibco, USA) No. 1 microscope cover glasses (VWR, USA), which, after 72 h in culture, were fixed in 4% PFA (Thermo Fisher Scientific, USA). Next, the same staining protocol was followed as for siRNA. All data are derived from at least three independent experiments (biological replicates), where each replicate contains 5–15 technical replicates. Data are reported as the mean with the corresponding standard deviation.

### Mitotic stage analysis

To quantify the percentage of telophase cells following siRNA treatment, HeLa-wt cells transfected with non-targeting-siRNA control, various siRNA constructs, or rescued with Rab11a-GFP or Rab35-GFP were fixed and stained using an anti-acetylated-α-tubulin antibody (Cell Signalling, USA, #D20G3, IF-1:200) and DAPI (Thermo Fisher Scientific, USA, #D1306), as described above. Cells in telophase were identified based on a mitotic spindle and chromatin condensation status. Cells were counted in 10 randomly selected fields in each independent experiment and expressed as a percentage of all cells. All data are derived from at least three independent experiments (biological replicates), where each replicate contains 5–15 technical replicates. Results are shown as mean ± standard deviation.

To quantify the percentage of telophase cells in shRNA-treated cells, stable cell lines with individual depletion of Rab11a, Rab11b or Rab35 were subjected to the same staining protocol used for siRNA experiments. All data are derived from at least three independent experiments (biological replicates), where each replicate contains 5–15 technical replicates. Results are shown as mean ± standard deviation.

### Actin fluorescence intensity measurements

The cells in late telophase, which had entirely reformed their cytoskeleton and no longer possessed a cleavage furrow, were selected for measuring the amount of actin at the ICB in both siRNA- and shRNA-treated cells. A single-image plane with a cell in late telophase, which includes the entire ICB in focus, was selected to measure F-actin fluorescence at the ICB. The regions of interest (ROIs) were then selected at the ICB to analyse F-actin enrichment. For the control, ROIs were selected at the opposing poles of the cell. Fluorescence intensity was then measured as a sum-fluorescence and normalised to the ROI size. Lastly, the ratio of fluorescence in the ICB and opposing poles was calculated to measure the enrichment of the F-actin at the ICB. The enrichment of actin fluorescence was counted in 10 carefully selected cytokinetic ICBs in each independent experiment (biological replicate) and expressed as a percentage change compared to the control (non-targeting-siRNA-control or non-targeting-shRNA-control). All data are derived from at least three independent experiments (biological repeats), with ten technical replicates for each biological repeat. Results are shown as mean ± standard deviation.

### Statistical analysis

Unless otherwise indicated, all data are derived from at least three independent experiments (biological replicates), calculating the mean and standard deviation. The data were processed using the Microsoft Office Excel data analysis tool pack (Microsoft Corporation, Redmond, WA, USA). A Student's *t*-test was used on all datasets to determine the significance level. Whenever three or more groups were compared, One-way ANOVA was used to perform statistical analysis. The level of significance was set as p < 0.05.

Data were collected from at least five randomly chosen image fields per coverslip for the multinucleation assay, mitotic stage analysis, and actin fluorescence intensity measurement. All the experiments were repeated at least three times (technical replicates). To quantify immunofluorescence, all specimens in the experiment were imaged using the same exposure settings, and image data were analysed using ImageJ software (National Institutes of Health, USA).

## Results

### The interrelationship between Rab11a, Rab11b, and Rab35 influences cytokinesis

**Fig. 1:**
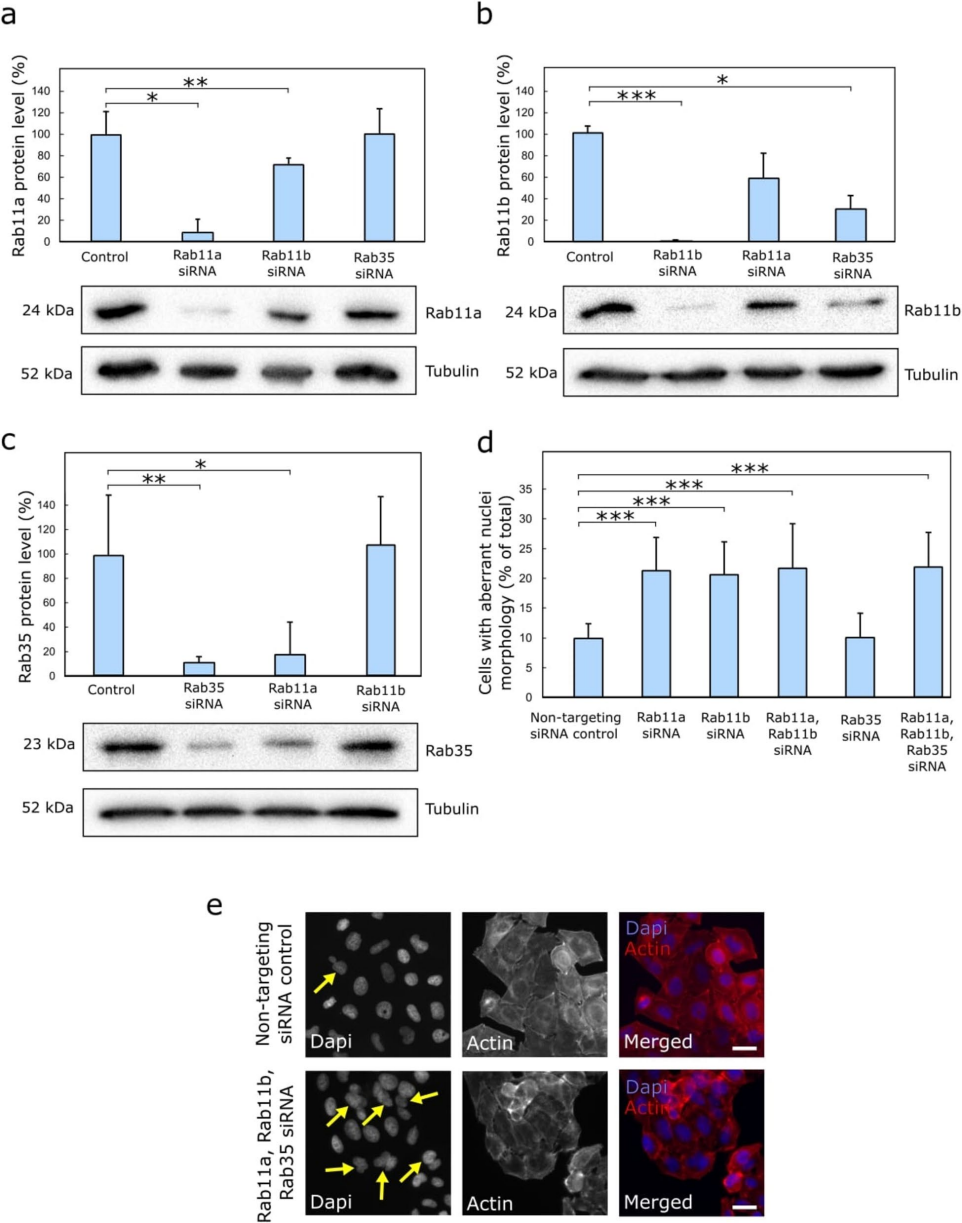
Silencing Rab11a, Rab11b, and Rab35 via siRNA induces multinucleation. **A**–**C** HeLa-wt cells transfected with different siRNA were cultured until confluency. Lysates were then collected, and the remaining expression of Rab11a, Rab11b and Rab35 proteins was measured using Western blotting. Relative protein quantification for cells treated with Rab11a, Rab11b, and Rab35 siRNAs is presented above the corresponding Western Blots. β-tubulin was used as a loading control, and the relative expression of the proteins was normalised to the level of intracellular tubulin. The data shown are the means and S.D. derived from at least three independent experiments. Asterisks (*) indicate statistically significant differences (p < 0.05). **D** Control and various siRNA-treated HeLa-wt cells were fixed and stained with DAPI and phalloidin, and the total number of binucleated, poly-lobed and micronucleated cells quantified. Asterisks (*) indicate statistically significant differences (p < 0.05). The data shown are the means and S.D. derived from at least three independent experiments. **E** The images depict HeLa-wt cell populations treated with non-targeting siRNA control and combined siRNAs, showing binucleated, poly-lobed, and micronuclei-containing cells (scale bar = 20 µm). Arrows highlight binucleated, poly-lobed, and micronucleated cells resulting from cytokinesis failure. A minimum of 30 images per group were taken using 20 × magnification. A minimum of three independent experiments were conducted in total

First, we used the RNA interference (RNAi) approach to downregulate Rab11a, Rab11b and Rab35 using different siRNAs. As expected, having almost 90% sequence identity, the downregulation of Rab11b by siRNA led to a reduction in Rab11a protein level by 28.1%, which is shown by WB analysis (Fig. 1A). These findings were consistent with the shRNA interference results, where Rab11b depletion resulted in a 97.4% reduction in Rab11a protein levels (Supplemental Fig S1A). Conversely, Rab11a downregulation by siRNA led to a 42.1% reduction in intracellular Rab11b levels compared to the control (Fig. 1B). This finding aligns with the shRNA data, where Rab11a depletion reduced Rab11b expression levels by 32.4% (Supplemental Fig S1B). Taken together, the data suggest a potential interrelation between the protein levels of Rab11 isoforms in vitro, where reduced expression of Rab11a may influence Rab11b levels, and vice versa. In contrast, neither Rab35 downregulation via siRNA nor its depletion through shRNA had a notable effect on Rab11a protein levels (Fig. 1A and Supplemental Fig S1A). However, unlike Rab11a, Rab11b protein levels were notably reduced following downregulation of Rab35, dropping to 29.8% with siRNA treatment and 5.4% with shRNA (Fig. 1B and Supplemental Fig S1B). Controversially, Rab35 protein levels decreased to 18.5%, following Rab11a downregulation via siRNA, whereas they remained almost unchanged at 102.7% in shRNA-treated cells (Fig. 1C and Supplemental Fig S1C). More confusing, following Rab11b siRNA treatment, Rab35 protein levels rose to 108.2% relative to the control (Fig. 1C). However, they decreased to 71.8% in cells treated with Rab11b shRNA (Supplemental Fig S1C).

The WB results above correspond well with the localisation data, where we used stable cell lines individually depleted of Rab11a, Rab11b, and Rab35 to examine the localisation of the other functionally similar Rab proteins. Under normal conditions, Rab11a, Rab11b, and Rab35 localise to both the entry points and the body of the ICB during telophase–characterised by a short, thick ICB–and during cytokinesis, when the ICB becomes long and narrow (Supplemental Fig S2A-C). However, when Rab11a is downregulated, the amount of Rab11b at the ICB during these late phases of the cell cycle is modestly decreased compared to control conditions (Supplemental Fig S2B). A comparable pattern is observed in Rab11b-depleted cells, where Rab11a levels at the ICB are also reduced (Supplemental Fig S2A). Again, this data suggests a potential interrelation between the protein levels of different Rab11 isoforms in vitro*.* In contrast, no localisation-based interrelation was observed between the Rab11 isoforms and Rab35, as Rab35 knockdown did not alter the localisation patterns of Rab11a or Rab11b (Supplemental Fig S2A-B). Likewise, Rab35 localisation was not significantly affected by the downregulation of either Rab11a or Rab11b (Supplemental Fig S2C).

Collectively, these findings suggest a possible in vitro interdependence between Rab11 isoform protein levels, where diminished expression of Rab11a may impact Rab11b levels, and vice versa (Fig. 1A, B, Supplemental Fig S1A-B, and Supplemental Fig S2A-B). The substantial reduction in Rab11b levels following Rab35 depletion via siRNA or shRNA suggests a potential connection between these two proteins; however, the nature of this relationship remains unclear at this stage (Fig. 1B and Supplemental Fig S1B). A similar uncertainty applies to Rab35 expression profiles following the downregulation of Rab11 isoforms, as the results appear inconsistent (Fig. 1C and Supplemental Fig S1C). If required, detailed information on the expression levels of Rab11 and Rab35, both under normal conditions and following downregulation, is available in the following research publications [16, 17, 21, 37].

Next, we fixed HeLa-wt cells transfected with either a non-targeting siRNA control or various siRNAs, followed by staining with phalloidin and DAPI. This allowed us to visualise cell nuclei (in blue) and delineate the cell cortex with red phalloidin-568 staining (Fig. 1E). Under normal physiological conditions, the HeLa-wt population in vitro contains approximately 5% abnormal mitotic and multinucleate cells [41]. In comparison, our study found that 9.9% (Fig. 1D) of the total population of HeLa-wt cells treated with a non-targeting siRNA contained abnormal nuclear morphology. Next, we observed that siRNA-mediated downregulation of either Rab11a or Rab11b resulted in a significant increase in abnormal nuclei morphology, observed in 21.3% and 20.6% of the total cell population, respectively (Fig. 1D). Meanwhile, simultaneous siRNA-mediated depletion of both Rab11a and Rab11b led to aberrant nuclear morphology in 21.8% of cells (Fig. 1D). Additionally, combined siRNA depletion of Rab11a, Rab11b, and Rab35 disrupted nuclear morphology in 22.0% of cells (Fig. 1D, E). This represented more than a twofold increase compared to the control (Fig. 1D). Notably, transfection with a plasmid encoding siRNA-resistant GFP-Rab11a resulted in a statistically significant and complete rescue of the cytokinetic defects caused by Rab11a downregulation in cancer cells (Supplemental Fig S3A). It is worth highlighting that the cytokinetic defects of Rab11a-siRNA-treated cells were also statistically significantly rescued by overexpression of siRNA-resistant GFP-Rab35, restoring them to control levels (Supplemental Fig S3A). The rescue effect of GFP-Rab11a on Rab11a-siRNA-downregulated was more pronounced than observed with GFP-Rab35 (Supplemental Fig S3A). Meanwhile, as Rab35 downregulation via RNAi did not result in a statistically significant change in nuclear morphology compared to the control (Fig. 1D), the rescue effects observed with siRNA-resistant GFP-Rab35 and GFP-Rab11a plasmids were negligible (Supplemental Fig S3B).

To further validate our siRNA-based findings on nuclear morphology, we analysed shRNA-treated cells with sustained downregulation of the proteins of interest. We found that shRNA-mediated knockdown of Rab11a and Rab11b had a pronounced impact on cells, with 27.0% and 29.9% of the total population exhibiting aberrant nuclear morphology (Supplemental Fig S1D). The knockdown effect of Rab35 using shRNA resulted in 13.3% (Supplemental Fig S1D) of cells displaying abnormal nuclear morphology, slightly higher than the 10.1% observed with siRNA (Fig. 1D), and statistically significantly greater than the 9.1% seen in the control (Supplemental Fig S1D).

Additionally, we conducted a WB analysis on individual Rab proteins to rule out potential off-target effects from double or even triple siRNA knockdown treatments that might influence the expression of the proteins of interest. We examined the expression levels of each Rab protein under combined double or triple knockdown conditions. The results showed that none of the experimental treatments affected the expression levels of the individual Rab proteins. Despite this, knockdowns effectively induced phenotypic changes in those cancer cells (Supplemental Fig S4A-C).

### The involvement of Rab11 and Rab35 in telophase progression and actin clearance from the intercellular bridge during cytokinesis

**Fig. 2:**
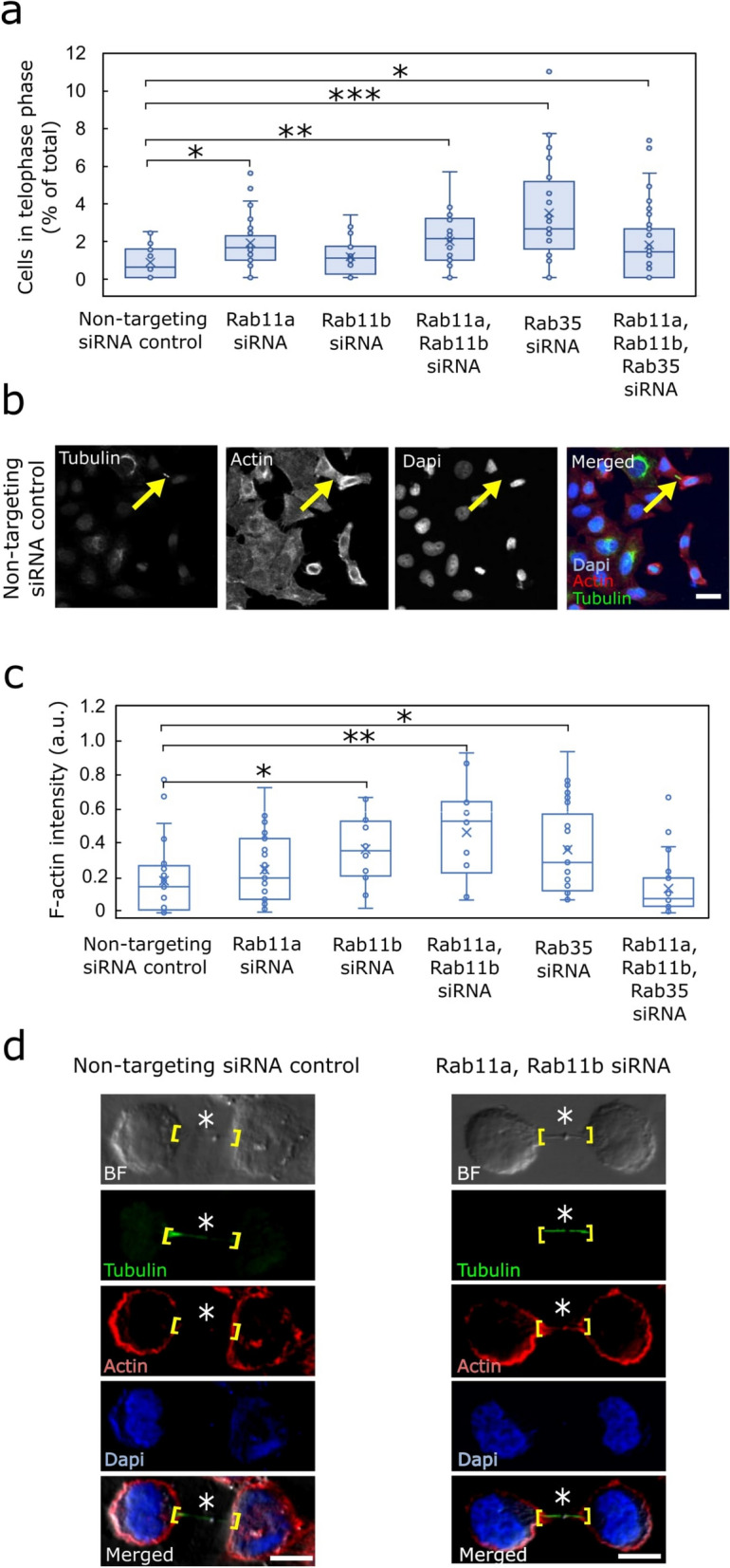
The downregulation of Rabs via siRNA causes telophase arrest or delay in more cells, accompanied by elevated F-actin at the intercellular bridge. **A** HeLa-wt cells were transfected with various siRNAs, then fixed and stained with DAPI, phalloidin, and an anti-acetylated-α-tubulin primary antibody. The number of cells in telophase was manually counted. Asterisks (*) indicate statistically significant differences (p < 0.05). At least 30 images were examined for each study group using 20 × magnification. A minimum of three independent experiments were conducted in total. **B** The images show HeLa-wt cells in telophase treated with a non-targeting siRNA control, with the ICB marked by an anti-acetylated-α-tubulin primary antibody (scale bar = 20 µm). Arrows indicate the ICB formed during cytokinesis. At least 30 images were examined for each study group using 20 × magnification. A minimum of three independent experiments were conducted in total. **C** The cells were prepared as specified in **A**. Actin enrichment at the ICB was then assessed by confocal microscopy using a 60 × oil immersion objective. The actin levels at the ICB were quantified by comparing control cells to those treated with individual siRNAs or combinations of siRNAs. Asterisks (*) indicate statistically significant differences (p < 0.05). For each study group, at least 10 different cells were examined. A minimum of three independent experiments were conducted in total. **D** siRNA-treated cells that failed to clear accumulated actin showed elevated actin levels at the ICB compared to controls (scale bar = 10 µm). The location of the ICB formation and the area where F-actin levels were measured are indicated in brackets. Asterisks (*) mark the midbody. Each study group examined at least 30 individual cells using 60 × magnification. A minimum of three independent experiments were conducted in total

Next, we fixed cells to evaluate whether Rab knockdown influences the proportion of cells arrested in telophase. We then performed staining with phalloidin 568, a marker for F-actin, and an anti-acetylated-α-tubulin antibody to label the ICB. This staining combination enabled clear visualisation of cells in telophase, characterised by the presence of an ICB between the dividing daughter cells (Fig. 2B and Supplemental Fig S5C). Among all siRNA-treated groups, Rab35 downregulation had the most pronounced effect on the number of cells in telophase. Specifically, 3.5% of the Rab35 siRNA-treated population were delayed or arrested in telophase, compared to just 0.8% of the non-targeting siRNA control (Fig. 2A), a difference also evident in the fluorescent images (Fig. 2B and Supplemental Fig S5C). Although the differences between the control and the other knockdown groups were statistically significant (apart from Rab11b siRNA), they were less substantial (Fig. 2A). Similar telophase delay or arrest patterns were also observed in various Rabs shRNA-treated cells. Among all groups, only Rab35 knockdown via shRNA resulted in a statistically significant increase, with 4.2% of cells affected compared to 1.8% in the control group (Supplemental Fig S6A).

Another key objective of this assay was to evaluate whether overexpression of different Rab proteins could rescue the observed phenotypic defects. The results showed that only the rescue of Rab35-depleted cells with siRNA-resistant GFP-Rab35 resulted in a statistically significant decrease in the number of cells delayed or arrested in telophase (Supplemental Fig S5A-B).

Following this, we quantified the levels of filamentous actin at the ICB during late telophase – a critical factor influencing cell separation, as excessive actin accumulation can hinder efficient ICB abscission. Late-stage ICBs are readily distinguishable, as they appear long and narrow just before the final abscission step (Fig. 2D, BF and tubulin-labelled images), making them easily severable. To quantify F-actin levels and enable comparison across different experimental groups, control and various siRNA-treated cells were processed for confocal microscopy, as described in the Methods section. In brief, high-magnification confocal microscopy (60 × oil immersion lens) was used to image the late-stage ICBs between dividing cells. To evaluate F-actin levels at the ICB, corrected total cell fluorescence (CTCF) [42] was calculated and normalised to background intensity, followed by determining the ratio of F-actin enrichment at the ICB relative to the cell pole.

F-actin accumulation was measured separately in Rab11a- and Rab11b-siRNA-treated cells, and in cells with simultaneous downregulation of both Rab11 isoforms—Rab11a and Rab11b (Fig. 2C). In these experiments, normalised F-actin levels at the cleavage furrow were measured at 0.26, 0.37, and 0.47 relative units in Rab11a-, Rab11b-, and dual Rab11a/Rab11b-siRNA-treated cells, respectively. All values–except Rab11a siRNA–were statistically significantly elevated compared to the 0.24 relative units observed in control cells treated with non-targeting siRNA (Fig. 2C). A comparable accumulation of F-actin at the ICB between the newly forming daughter cells was also observed in Rab35 siRNA-treated cells (Fig. 2C). F-actin enrichment in this group reached 0.37 relative units, representing a statistically significant 54.2% increase compared to the control. However, this trend did not persist with the triple knockdown of Rab11a, Rab11b, and Rab35, where F-actin levels were dropped to a meaningless 0.15 relative units (Fig. 2C). In comparison, shRNA-mediated knockdown of individual Rab proteins produced a similar progressive pattern of F-actin accumulation as observed with siRNA, but the effect was much more pronounced. Compared to the F-actin level at 0.28 relative units in the non-targeting shRNA control, these levels increased markedly to 4.24, 4.79, and 6.26 relative units in Rab11a-, Rab11b, and Rab35-shRNA-depleted cells, respectively, with all increases being statistically significant relative to the control (Supplemental Fig S6B).

**Fig. 3:**
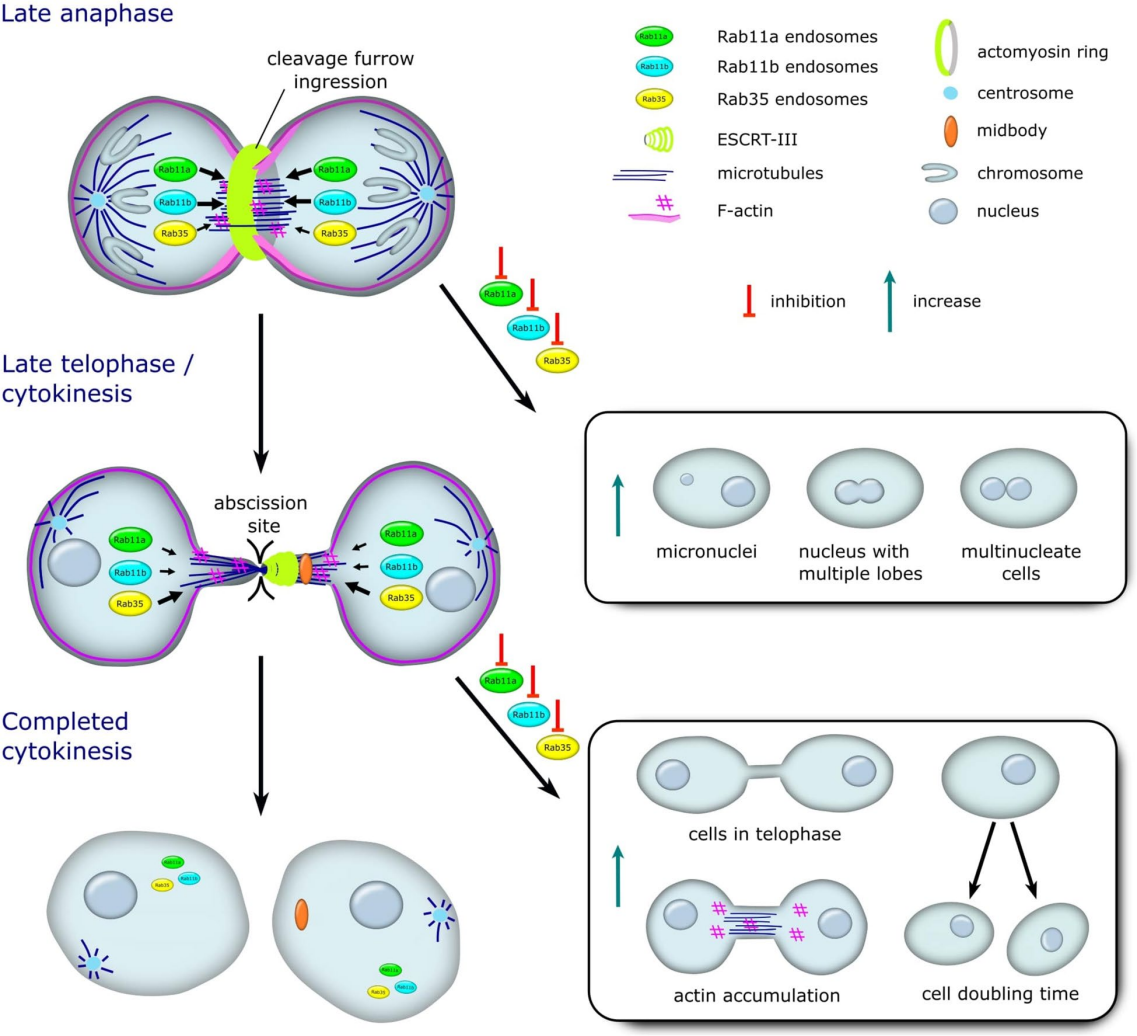
A model illustrating the roles of Rab11 and Rab35 during the transition from late anaphase to complete cytokinesis. On the left side of the image, the size of arrows next to the endosome illustration reflects the relative importance and involvement of each Rab protein in distinct phases of the cell cycle. The right side illustrates the cellular processes affected by Rab protein inhibition during cleavage furrow ingression and abscission site formation

In summary, the collective results presented above suggest that Rab11 and Rab35 are critical regulators of cytoskeletal remodelling during the final phases of cell division. Notably, disruption of this process can impair actin clearance from the ICB, potentially hindering its proper severing at the abscission site during cytokinesis. This failure in mitotic exit can lead to nuclear abnormalities, such as polyploidy and tetraploidy, which–if left unchecked–may contribute to the development of tumorigenic phenotypes (Fig. 3) [14].

## Discussion

Cell division is a complex and tightly regulated process requiring coordinated cytoskeleton remodelling and intracellular transport to separate the newly formed daughter cells into two distinct entities. Specifically, Rab11 and Rab35 have been shown to facilitate endocytic transport to the division site in cells that are either preparing to complete division or actively undergoing the physical separation process [43, 44].

In this study, we discovered that downregulation of individual Rab11 isoforms may affect the expression levels of the remaining Rab11 isoforms still present in the cells (Fig. 1A, B and Supplemental Fig S1A-B). This was further supported by the localisation patterns of Rab11a and Rab11b observed following the downregulation of either isoform (Supplemental Fig S2A-B). These findings were anticipated by the high sequence homology among Rab11 isoforms [45]. Unfortunately, despite our efforts, we were unable to establish any interrelation between the expression levels of Rab11 isoforms and Rab35 (Fig. 1A–C and Supplemental Fig S1A-C), nor could we clearly determine the localisation patterns of either protein in cells depleted of Rab11a-, Rab11b-, and Rab35 using shRNA (Supplemental Fig S2A-C). The results were inconsistent and inconclusive, despite employing both siRNA and shRNA approaches to downregulate Rab proteins, making it difficult to draw any definitive conclusions.

Rab11 and Rab35 recycling endosomes have been identified as central regulators of the targeted disassembly of the locally accumulated F-actin network at the ICB during cytokinesis [18]. Building on this, we next aimed to investigate how the downregulation of individual or combined Rab proteins might influence cleavage furrow formation during late anaphase (Fig. 3). Downregulation of Rab11a or Rab11b using siRNA resulted in over 20% of the total cell population exhibiting aberrant nuclear morphology (Fig. 1D). This phenotype was even more pronounced in Rab11a- and Rab35-shRNA-treated cells, with nearly 30% of the total population containing aberrant nuclear morphology (Supplemental Fig S1D). Additionally, our results showed that co-depletion of Rab11a and Rab11b did not significantly enhance phenotypic defects associated with their individual downregulation during cleavage furrow formation (Fig. 1D). In comparison, *Yu* et al. employed a different strategy by transfecting HeLa cells with a dominant-negative Rab11 mutant, which led to a comparable increase in multinucleation to approximately 15% [9]. Additionally, siRNA-mediated depletion of either Rab11a or Rab11b resulted in the formation of binucleated cells at a rate of approximately 13% [9]. Meanwhile, Rab35 downregulation did not lead to a significant increase in cells exhibiting aberrant nuclear morphology, with levels remaining comparable to the control, approximately 10% in siRNA-treated cells and 13% in shRNA-treated cells (Fig. 1D and Supplemental Fig S1D). For comparison, a similar study using *Drosophila* as a model system showed that Rab35 downregulation via RNAi led to an increase in binucleation to approximately 5% and 20% at third and sixth days post-transfection, respectively [21]. Indeed, our results were obtained 3 days following transfection with the corresponding siRNAs. Unfortunately, simultaneous triple knockdown of Rab11a, Rab11b, and Rab35 using siRNAs did not produce an additive effect on cleavage furrow formation defects, with only 22.0% of cells exhibiting aberrant nuclear morphology (Fig. 1D). Finally, the phenotypic defects associated with Rab11a downregulation during cleavage furrow formation could be reversed by overexpressing either the same gene or a functionally related Rab protein (Supplemental Fig S3A). Altogether, the data suggest the existence of a molecular interplay or functional interrelationship between different Rab11 isoforms.

Next, we aimed to investigate whether Rab protein downregulation impacts the cell's ability to transition from late anaphase to telophase/cytokinesis. This critical step determines the success of cell division. To address this, we assessed the number of cells in telophase following Rab protein downregulation. According to the results, Rab35 emerged as the most likely key contributor, as its depletion led to a marked increase in the number of cells being delayed or arrested in telophase—rising to 3.5% with siRNA treatment, over four times higher than the non-targeting siRNA contol (Fig. 2A), and to 4.2% with shRNA treatment, more than double that of the non-targeting shRNA control (Supplemental Fig S6A). Yet it is important to acknowledge that neither individual nor combined downregulation of the other Rabs caused a significant change, as the proportion of cells delayed or arrested in telophase remained close to approximately 2% (Fig. 2A and Supplemental Fig S6A).

Based on the results from the two primary assays–one examining the transition from late anaphase to late telophase/cytokinesis, and the other evaluating progression from late telophase/cytokinesis to completed cell division–it can be speculated that Rab11a and Rab11b play a more prominent role in driving cleavage furrow ingression during late anaphase. In contrast, Rab35 appears to have a lesser role in cleavage furrow formation but is more essential for establishing the abscission site at the ICB (Fig. 3). In comparison, *Dambournet* et al. demonstrated that Rab35 knockdown prolongs the duration of the final abscission step, directly influencing the ultimate fate of dividing cells [22].

To underscore Rab35’s critical role in completing cytokinesis, *Dambournet* et al. also demonstrated that depletion of either Rab35 or its effector OCRL leads to elevated levels of PtdIns(4,5)P_2_ and F-actin accumulation at the ICB [22]. In many studies investigating the mechanisms of cell division, the failure to depolymerise F-actin at the abscission site within the ICB is frequently identified as a primary cause of unsuccessful cytokinesis. In line with previous findings, our study also demonstrated that siRNA-mediated depletion of Rab11b, or Rab35 individually, as well as those with combined Rab11a and Rab11b siRNA depletion, exhibited difficulties in clearing F-actin from the mitotic ICB (Fig. 2C, D). These findings were further supported by shRNA data, which revealed an even greater accumulation of F-actin levels across all Rab shRNA-treated cell groups compared to the control, with the most pronounced effect observed in Rab35-shRNA-treated cells (Supplemental Fig S6B). These findings suggest that reduced expression of Rab proteins indeed impairs actin clearance from the abscission site, thereby compromising the successful execution of cell division. For comparison, in one of our previous studies, we demonstrated that another member of the Rab family, Rab14, also plays a role in actin clearance at the abscission site. Specifically, CRISPR-mediated knockout of Rab14 impaired cytokinesis and increased F-actin accumulation at the mitotic ICB [46], mirroring the effects observed in Rab11a- and Rab35-siRNA-treated cells in this study.

## Conclusions

In summary, the results suggest a functional interrelationship between Rab11 isoforms, as the downregulation of Rab11a appears to affect the expression levels of Rab11b, and vice versa. Although Rab11 and Rab35 were identified as key regulators of cell division, our study clarifies the specific and distinct roles each protein plays during mitosis. The Rab11 isoforms likely play a more prominent role in initiating cleavage furrow ingression during late anaphase, whereas it is plausible that Rab35 serves as the key regulator in establishing the abscission site during the late telophase and cytokinesis. The results suggest that cytokinetic defects resulting from Rab11 or Rab35 downregulation are linked to irregular accumulation of F-actin at the ICB. Importantly, although intracellular protein levels can fluctuate during gene manipulation studies, the simultaneous depletion of Rab11 and Rab35 did not produce any additive effects on cytokinesis-related defects. Therefore, the mechanism for molecular interrelation between Rab11 and Rab35 remains inconclusive and requires further investigation.

## Supplementary Information

Supplementary material 1: Figure 1. Silencing Rab11a, Rab11b, and Rab35 via shRNA induces multinucleation.Stable cell lines expressing various shRNAs were cultured to confluency. Cell lysates were collected, and the remaining levels of Rab11a, Rab11b, and Rab35 proteins after shRNA treatment were analysed by Western blotting. Quantification of protein expression in Rab11a-, Rab11b-, and Rab35-depleted cells is shown above each corresponding blot. β-tubulin served as a loading control, and protein levels were normalised to intracellular tubulin expression.Control and shRNA-treated stable cells were fixed and stained with DAPI and phalloidin. The numbers of binucleated, poly-lobed, and micronucleated cells were manually quantified. Asterisks (*) indicate statistically significant differences. The data shown are the means and S.D. derived from at least three independent experiments.

Supplementary material 2: Figure 2. Localisation of Rab11a, Rab11b, and Rab35 during telophase and cytokinesis in control and shRNA-depleted cells.The localisation of Rab11a, Rab11b, and Rab35 was examined in stable cell lines treated with either non-targeting shRNA control or individually depleted of each Rab protein using specific shRNAs. Briefly, stable cell lines expressing different shRNAs were fixed and stained with DAPI, an anti-acetylated-α-tubulin antibody, and a specific antibody against either Rab11a, Rab11b, or Rab35. The localisation pattern relative to tubulin at the ICB was analysed in telophase and cytokinesis. Asterisks (*) indicate Rab protein localisation at the ICB in the corresponding cell line.

Supplementary material 3: Figure 3. Reversal of phenotypic changes caused by Rab11a, Rab11b, and Rab35 silencing.HeLa-wt cells treated with non-targeting siRNA control or specific siRNAs were subsequently transfected with siRNA-resistant Rab11a-GFP or Rab35-GFP plasmids, followed by fixation and staining with DAPI and phalloidin. Successful cell division was assessed by quantifying the total number of multinucleated cells, including those that were binucleated, poly-lobed, or contained micronuclei. Results were expressed as the percentage of total cells counted. The image shows the rescue of the KD with the same gene or overexpression of a different gene. Asterisks (*) indicate statistically significant differences. The data shown are the means and S.D. derived from at least three independent experiments. Unless stated otherwise, at least 30 images per study group were acquired using 20× magnification.

Supplementary material 4: Figure 4. The expression of Rab11a, Rab11b and Rab35 remains unaffected by double or triple knockdown of Rab proteins.HeLa-wt cells transfected with individual or combined siRNAs were cultured to confluency. Cell lysates were then collected, and the relative protein levels of Rab11a, Rab11b, or Rab35 were assessed by Western blot analysis. Quantification of each is shown above the corresponding WB bands. β-tubulin served as a loading control, and protein expression levels were normalised to intracellular tubulin

Supplementary material 5: Figure 5. Restoring normal telophase progression following Rab11a, Rab11b, and Rab35 depletion.HeLa-wt cells treated with non-targeting siRNA control or specific siRNAs were subsequently transfected with siRNA-resistant Rab11a-GFP or Rab35-GFP plasmids, followed by fixation and staining with DAPI, phalloidin, and anti-acetylated-α-tubulin primary antibody. The telophase phase count was then performed manually. The image shows the rescue of the KD with the same gene or overexpression of a different gene. Asterisks (*) indicate statistically significant differences. The data shown are the means and S.D. derived from at least three independent experiments. Unless otherwise stated, at least 30 images per study group were acquired using 20× magnification.The images depict an increased number of cells being delayed or arrested in telophase following Rab35 siRNA treatment. This was identified by staining the ICB with an anti-acetylated-α-tubulin primary antibody. Arrows highlight various ICBs formed during late telophase.

Supplementary material 6: Figure 6. The downregulation of Rabs via shRNA causes telophase arrest or delay in more cells, accompanied by elevated F-actin at the intercellular bridge.Stable cell lines expressing different shRNAs were fixed and stained with DAPI, phalloidin, and an anti-acetylated-α-tubulin primary antibody. The number of cells in telophase was manually counted. Asterisks (*) indicate statistically significant differences. At least 30 images were examined for each study group using 20× magnification. A minimum of three independent experiments were conducted in total. Results are shown as mean ± standard deviation.The cells were prepared as specified in Supplemental Fig 6A. Actin enrichment at the ICB was then assessed by confocal microscopy using a 60× oil immersion objective. The actin levels at the ICB were quantified by comparing control cells to those treated with individual shRNA. Asterisks (*) indicate statistically significant differences. For each study group, at least 10 different cells were examined. A minimum of three independent experiments were conducted in total. Results are shown as mean ± standard deviation.

## Data Availability

The datasets used and/or analysed during the current study are available from the corresponding author on reasonable request.

## References

[1] Carlton JG, Jones H, Eggert US. Membrane and organelle dynamics during cell division. Nat Rev Mol Cell Biol. 2020;21:151–66. 32034394 10.1038/s41580-019-0208-1

[2] Fededa JP, Gerlich DW. Molecular control of animal cell cytokinesis. Nat Cell Biol. 2012;14:440–7. 22552143 10.1038/ncb2482

[3] Schiel JA, Prekeris R. Membrane dynamics during cytokinesis. Curr Opin Cell Biol. 2013;25:92–8. 23177492 10.1016/j.ceb.2012.10.012 PMC4228953

[4] Chew TG, Huang J, Palani S, Sommese R, Kamnev A, Hatano T, et al. Actin turnover maintains actin filament homeostasis during cytokinetic ring contraction. J Cell Biol. 2017;216:2657–67. 28655757 10.1083/jcb.201701104 PMC5584170

[5] Pollard TD, O’Shaughnessy B. Molecular mechanism of cytokinesis. Annu Rev Biochem. 2019;88:661–89. 30649923 10.1146/annurev-biochem-062917-012530 PMC6588489

[6] Pollard TD. Mechanics of cytokinesis in eukaryotes. Curr Opin Cell Biol. 2010;22:50–6. 20031383 10.1016/j.ceb.2009.11.010 PMC2871152

[7] Miserey-Lenkei S, Colombo MI. Small RAB GTPases regulate multiple steps of mitosis. Front Cell Dev Biol. 2016;4:2. 26925400 10.3389/fcell.2016.00002 PMC4756281

[8] Nakayama K. Regulation of cytokinesis by membrane trafficking involving small GTPases and the ESCRT machinery. Crit Rev Biochem Mol Biol. 2016;51:1–6. 26362026 10.3109/10409238.2015.1085827

[9] Yu X, Prekeris R, Gould GW. Role of endosomal rab GTPases in cytokinesis. Eur J Cell Biol. 2007;86:25–35. 17157409 10.1016/j.ejcb.2006.10.002

[10] Saito S, Liu X-F, Kamijo K, Raziuddin R, Tatsumoto T, Okamoto I, et al. Deregulation and mislocalization of the cytokinesis regulator ECT2 activate the Rho signaling pathways leading to malignant transformation*. J Biol Chem. 2004;279:7169–79. 14645260 10.1074/jbc.M306725200

[11] Nguyen HG, Makitalo M, Yang D, Chinnappan D, St. Hilaire C, Ravid K. Deregulated Aurora-B induced tetraploidy promotes tumorigenesis. FASEB J. 2009;23:2741–8. 19332642 10.1096/fj.09-130963 PMC2717782

[12] Dong W, Wu X. Overexpression of Rab11-FIP2 in colorectal cancer cells promotes tumor migration and angiogenesis through increasing secretion of PAI-1. Cancer Cell Int. 2018;18:35. 29540997 10.1186/s12935-018-0532-0 PMC5845176

[13] Guadagno NA, Progida C. Rab GTPases: switching to human diseases. Cells. 2019;8:909. 31426400 10.3390/cells8080909 PMC6721686

[14] Fujiwara T, Bandi M, Nitta M, Ivanova EV, Bronson RT, Pellman D. Cytokinesis failure generating tetraploids promotes tumorigenesis in p53 -null cells. Nature. 2005;437:1043–7. 16222300 10.1038/nature04217

[15] Högnäs G, Tuomi S, Veltel S, Mattila E, Murumägi A, Edgren H, et al. Cytokinesis failure due to derailed integrin traffic induces aneuploidy and oncogenic transformation in vitro and in vivo. Oncogene. 2012;31:3597–606. 22120710 10.1038/onc.2011.527 PMC3419982

[16] Wilson GM, Fielding AB, Simon GC, Yu X, Andrews PD, Hames RS, et al. The FIP3-Rab11 protein complex regulates recycling endosome targeting to the cleavage furrow during late cytokinesis. Mol Biol Cell. 2005;16:849–60. 15601896 10.1091/mbc.E04-10-0927 PMC545916

[17] Fielding AB, Schonteich E, Matheson J, Wilson G, Yu X, Hickson GRX, et al. Rab11-FIP3 and FIP4 interact with Arf6 and the exocyst to control membrane traffic in cytokinesis. EMBO J. 2005;24:3389–99. 16148947 10.1038/sj.emboj.7600803 PMC1276165

[18] Gibieža P, Petrikaitė V. The dual functions of Rab11 and Rab35 GTPases-regulation of cell division and promotion of tumorigenicity. Am J Cancer Res. 2021;11:1861–72. 34094658 PMC8167671

[19] Schiel JA, Simon GC, Zaharris C, Weisz J, Castle D, Wu CC, et al. *FIP3*-endosome-dependent formation of the secondary ingression mediates ESCRT-III recruitment during cytokinesis. Nat Cell Biol. 2012;14:1068–78. 23000966 10.1038/ncb2577 PMC4495918

[20] Takahashi S, Takei T, Koga H, Takatsu H, Shin H-W, Nakayama K. Distinct roles of Rab11 and Arf6 in the regulation of Rab11-FIP3/arfophilin-1 localization in mitotic cells. Genes Cells. 2011;16:938–50. 21790911 10.1111/j.1365-2443.2011.01538.x

[21] Kouranti I, Sachse M, Arouche N, Goud B, Echard A. Rab35 regulates an endocytic recycling pathway essential for the terminal steps of cytokinesis. Curr Biol. 2006;16:1719–25. 16950109 10.1016/j.cub.2006.07.020

[22] Dambournet D, Machicoane M, Chesneau L, Sachse M, Rocancourt M, El Marjou A, et al. Rab35 GTPase and OCRL phosphatase remodel lipids and F-actin for successful cytokinesis. Nat Cell Biol. 2011;13:981–8. 21706022 10.1038/ncb2279

[23] Niu F, Sun K, Wei W, Yu C, Wei Z. F-actin disassembly factor MICAL1 binding to Myosin Va mediates cargo unloading during cytokinesis. Sci Adv. 2020;6:eabb1307. 33158857 10.1126/sciadv.abb1307 PMC7673715

[24] Frémont S, Romet-Lemonne G, Houdusse A, Echard A. Emerging roles of MICAL family proteins - from actin oxidation to membrane trafficking during cytokinesis. J Cell Sci. 2017;130:1509–17. 28373242 10.1242/jcs.202028

[25] Hung R-J, Pak CW, Terman JR. Direct redox regulation of F-actin assembly and disassembly by Mical. Science. 2011;334:1710–3. 22116028 10.1126/science.1211956 PMC3612955

[26] Vietri M, Schink KO, Campsteijn C, Wegner CS, Schultz SW, Christ L, et al. Spastin and ESCRT-III coordinate mitotic spindle disassembly and nuclear envelope sealing. Nature. 2015;522:231–5. 26040712 10.1038/nature14408

[27] Connell JW, Lindon C, Luzio JP, Reid E. Spastin couples microtubule severing to membrane traffic in completion of cytokinesis and secretion. Traffic Cph Den. 2009;10:42–56. 10.1111/j.1600-0854.2008.00847.x PMC2709849 19000169

[28] Caballe A, Martin-Serrano J. Escrt machinery and cytokinesis: the road to daughter cell separation. Traffic. 2011;12:1318–26. 21722282 10.1111/j.1600-0854.2011.01244.x

[29] Christ L, Wenzel EM, Liestøl K, Raiborg C, Campsteijn C, Stenmark H. ALIX and ESCRT-I/II function as parallel ESCRT-III recruiters in cytokinetic abscission. J Cell Biol. 2016;212:499–513. 26929449 10.1083/jcb.201507009 PMC4772496

[30] Carlton JG, Martin-Serrano J. Parallels between cytokinesis and retroviral budding: a role for the ESCRT machinery. Science. 2007;316:1908–12. 17556548 10.1126/science.1143422

[31] Elia N, Sougrat R, Spurlin TA, Hurley JH, Lippincott-Schwartz J. Dynamics of endosomal sorting complex required for transport (ESCRT) machinery during cytokinesis and its role in abscission. Proc Natl Acad Sci U S A. 2011;108:4846–51. 21383202 10.1073/pnas.1102714108 PMC3064317

[32] Morita E, Sandrin V, Chung H-Y, Morham SG, Gygi SP, Rodesch CK, et al. Human ESCRT and ALIX proteins interact with proteins of the midbody and function in cytokinesis. EMBO J. 2007;26:4215–27. 17853893 10.1038/sj.emboj.7601850 PMC2230844

[33] Gemperle J, Liße D, Kappen M, Secret E, Coppey M, Gregor M, et al. Live-cell magnetic manipulation of recycling endosomes reveals their direct effect on actin protrusions to promote invasive migration. Sci Adv. 2025;11:eadu6361. 40614209 10.1126/sciadv.adu6361 PMC12227070

[34] Barlow HR, Ahuja N, Bierschenk T, Htike Y, Fassetta L, Azizoglu DB, et al. Rab11 is essential to pancreas morphogenesis, lumen formation and endocrine mass. Dev Biol. 2023;499:59–74. 37172642 10.1016/j.ydbio.2023.05.002

[35] Zhu H, Xue C, Xu X, Guo Y, Li X, Lu J, et al. Rab8a/Rab11a regulate intercellular communications between neural cells via tunneling nanotubes. Cell Death Dis. 2016;7:e2523. 28005071 10.1038/cddis.2016.441 PMC5260982

[36] Ding T, Ji J, Zhang W, Liu Y, Liu B, Han Y, et al. The phosphatidylinositol (4,5)-bisphosphate-Rab35 axis regulates migrasome formation. Cell Res. 2023;33:617–27. 37142675 10.1038/s41422-023-00811-5 PMC10397319

[37] Klinkert K, Rocancourt M, Houdusse A, Echard A. Rab35 GTPase couples cell division with initiation of epithelial apico-basal polarity and lumen opening. Nat Commun. 2016;7:11166. 27040773 10.1038/ncomms11166 PMC4822036

[38] Chevallier J, Koop C, Srivastava A, Petrie RJ, Lamarche-Vane N, Presley JF. Rab35 regulates neurite outgrowth and cell shape. FEBS Lett. 2009;583:1096–101. 19289122 10.1016/j.febslet.2009.03.012

[39] Iannantuono NVG, Emery G. Rab11FIP1 maintains Rab35 at the intercellular bridge to promote actin removal and abscission. J Cell Sci. 2021;134:jcs244384. 34152390 10.1242/jcs.244384

[40] Chesneau L, Dambournet D, Machicoane M, Kouranti I, Fukuda M, Goud B, et al. An ARF6/Rab35 GTPase cascade for endocytic recycling and successful cytokinesis. Curr Biol. 2012;22:147–53. 22226746 10.1016/j.cub.2011.11.058

[41] Yasuda S, Taniguchi H, Oceguera-Yanez F, Ando Y, Watanabe S, Monypenny J, et al. An essential role of Cdc42-like GTPases in mitosis of HeLa cells. FEBS Lett. 2006;580:3375–80. 16716304 10.1016/j.febslet.2006.05.009

[42] Jakic B, Buszko M, Cappellano G, Wick G. Elevated sodium leads to the increased expression of HSP60 and induces apoptosis in HUVECs. PLoS ONE. 2017;12:e0179383. 28604836 10.1371/journal.pone.0179383 PMC5467851

[43] Frémont S, Hammich H, Bai J, Wioland H, Klinkert K, Rocancourt M, et al. Oxidation of F-actin controls the terminal steps of cytokinesis. Nat Commun. 2017;8:14528. 28230050 10.1038/ncomms14528 PMC5331220

[44] Laflamme C, Galan JA, Ben El Kadhi K, Méant A, Zeledon C, Carréno S, et al. Proteomics screen identifies class I Rab11 family interacting proteins as key regulators of cytokinesis. Mol Cell Biol. 2017;37:e00278-16. 27872148 10.1128/MCB.00278-16 PMC5247615

[45] Vale-Costa S, Amorim MJ. Recycling endosomes and viral infection. Viruses. 2016;8:64. 27005655 10.3390/v8030064 PMC4810254

[46] Gibieža P, Peterman E, Hoffman HK, Van Engeleburg S, Skeberdis VA, Prekeris R. Rab14/MACF2 complex regulates endosomal targeting during cytokinesis. Mol Biol Cell. 2021;32:554–66. 33566684 10.1091/mbc.E20-09-0607 PMC8101466

