# Supplementary figures and images for: The unique contributions of Rab11 and Rab35 to the completion of cell division
Source: Biol Res. 2025 Aug 29;58:59. doi: 10.1186/s40659-025-00638-x (PMC12395799; doi:10.1186/s40659-025-00638-x)

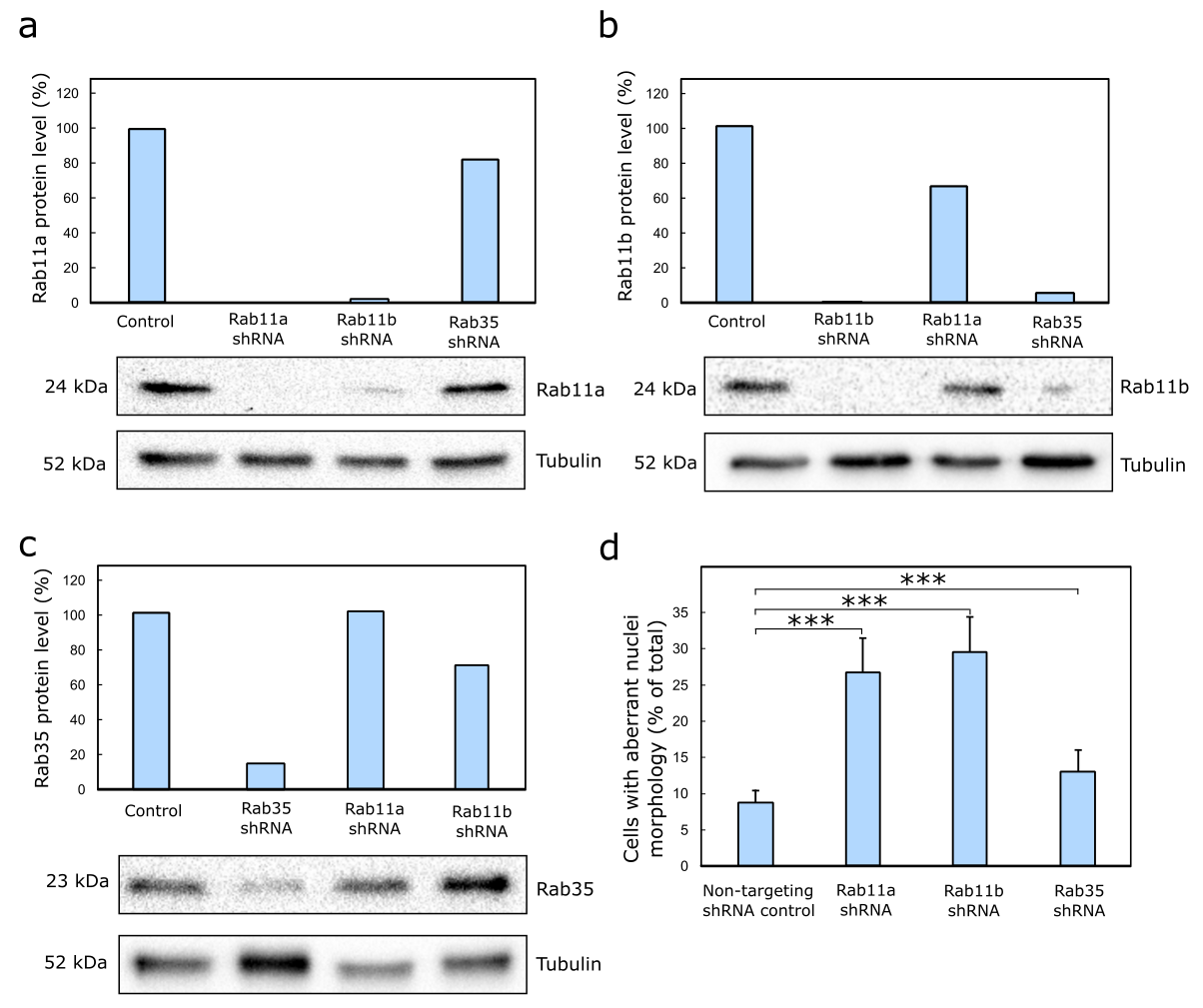

Supplement: Supplementary file 1 — Supplementary material 1: Figure 1. Silencing Rab11a, Rab11b, and Rab35 via shRNA induces multinucleation.Stable cell lines expressing various shRNAs were cultured to confluency. Cell lysates were collected, and the remaining levels of Rab11a, Rab11b, and Rab35 proteins after shRNA treatment were analysed by Western blotting. Quantification of protein expression in Rab11a-, Rab11b-, and Rab35-depleted cells is shown above each corresponding blot. β-tubulin served as a loading control, and protein levels were normalised to intracellular tubulin expression.Control and shRNA-treated stable cells were fixed and stained with DAPI and phalloidin. The numbers of binucleated, poly-lobed, and micronucleated cells were manually quantified. Asterisks (*) indicate statistically significant differences. The data shown are the means and S.D. derived from at least three independent experiments. [file 40659_2025_638_MOESM1_ESM.tiff]

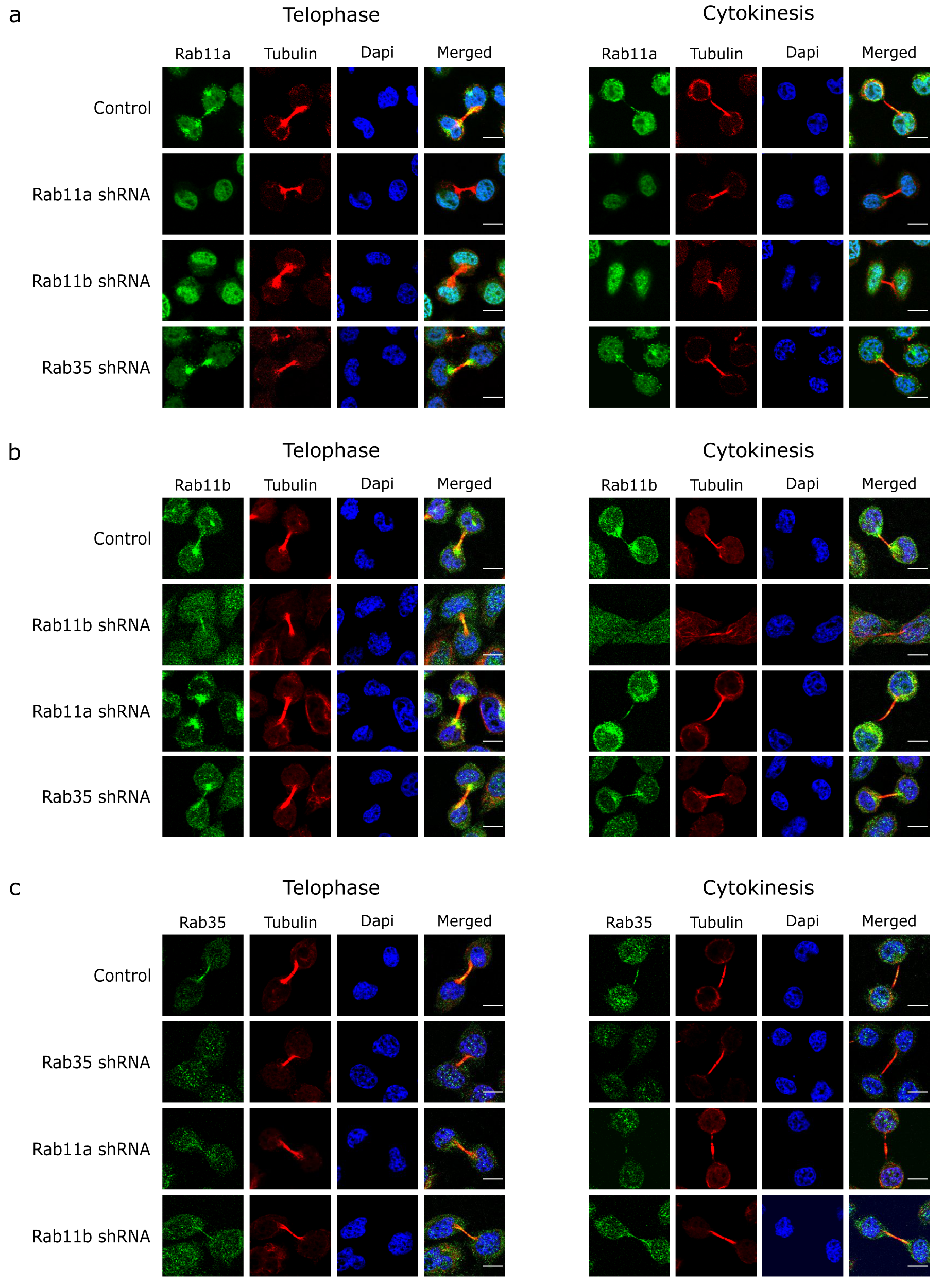

Supplement: Supplementary file 2 — Supplementary material 2: Figure 2. Localisation of Rab11a, Rab11b, and Rab35 during telophase and cytokinesis in control and shRNA-depleted cells.The localisation of Rab11a, Rab11b, and Rab35 was examined in stable cell lines treated with either non-targeting shRNA control or individually depleted of each Rab protein using specific shRNAs. Briefly, stable cell lines expressing different shRNAs were fixed and stained with DAPI, an anti-acetylated-α-tubulin antibody, and a specific antibody against either Rab11a, Rab11b, or Rab35. The localisation pattern relative to tubulin at the ICB was analysed in telophase and cytokinesis. Asterisks (*) indicate Rab protein localisation at the ICB in the corresponding cell line. [file 40659_2025_638_MOESM2_ESM.tiff]

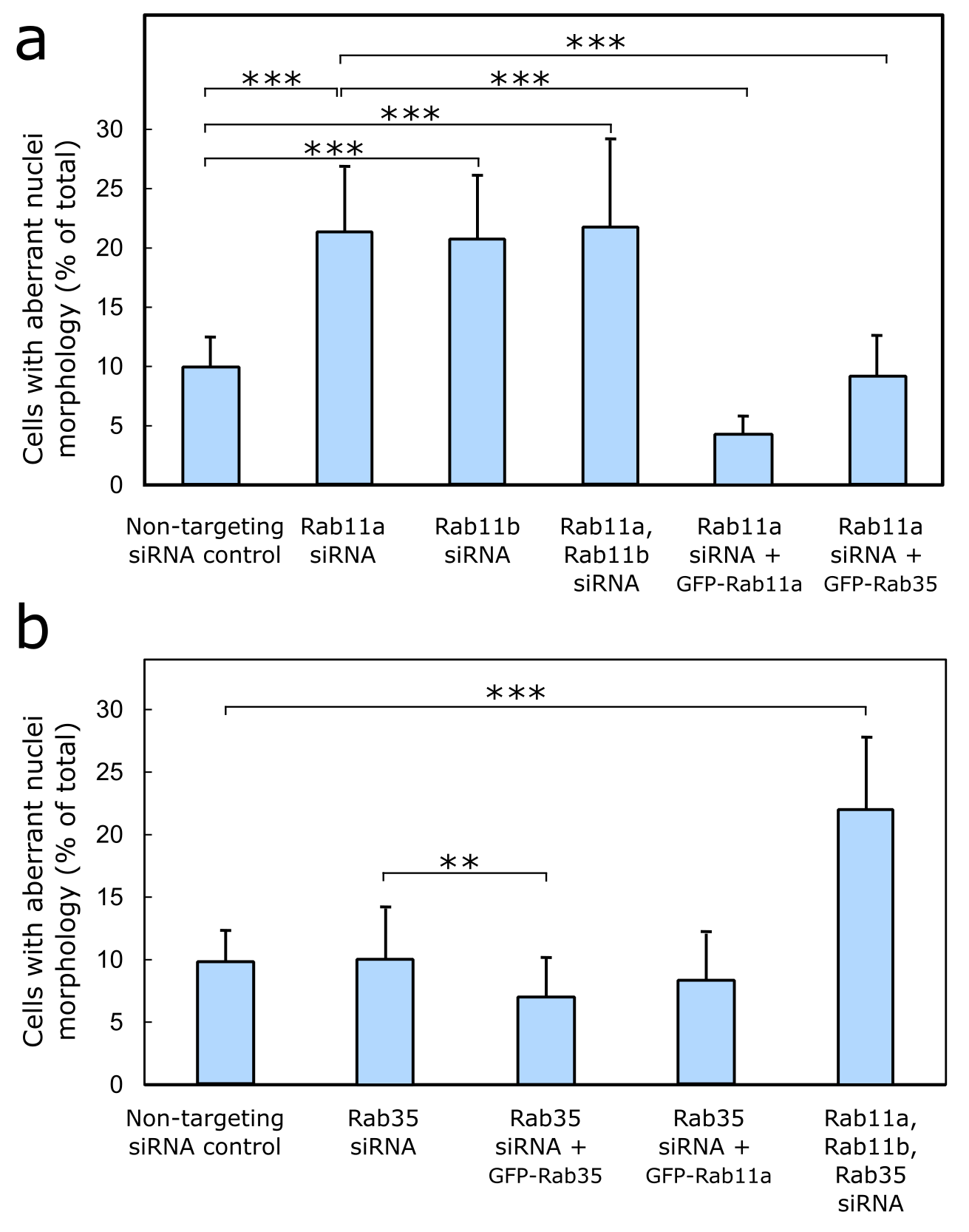

Supplement: Supplementary file 3 — Supplementary material 3: Figure 3. Reversal of phenotypic changes caused by Rab11a, Rab11b, and Rab35 silencing.HeLa-wt cells treated with non-targeting siRNA control or specific siRNAs were subsequently transfected with siRNA-resistant Rab11a-GFP or Rab35-GFP plasmids, followed by fixation and staining with DAPI and phalloidin. Successful cell division was assessed by quantifying the total number of multinucleated cells, including those that were binucleated, poly-lobed, or contained micronuclei. Results were expressed as the percentage of total cells counted. The image shows the rescue of the KD with the same gene or overexpression of a different gene. Asterisks (*) indicate statistically significant differences. The data shown are the means and S.D. derived from at least three independent experiments. Unless stated otherwise, at least 30 images per study group were acquired using 20× magnification. [file 40659_2025_638_MOESM3_ESM.tiff]

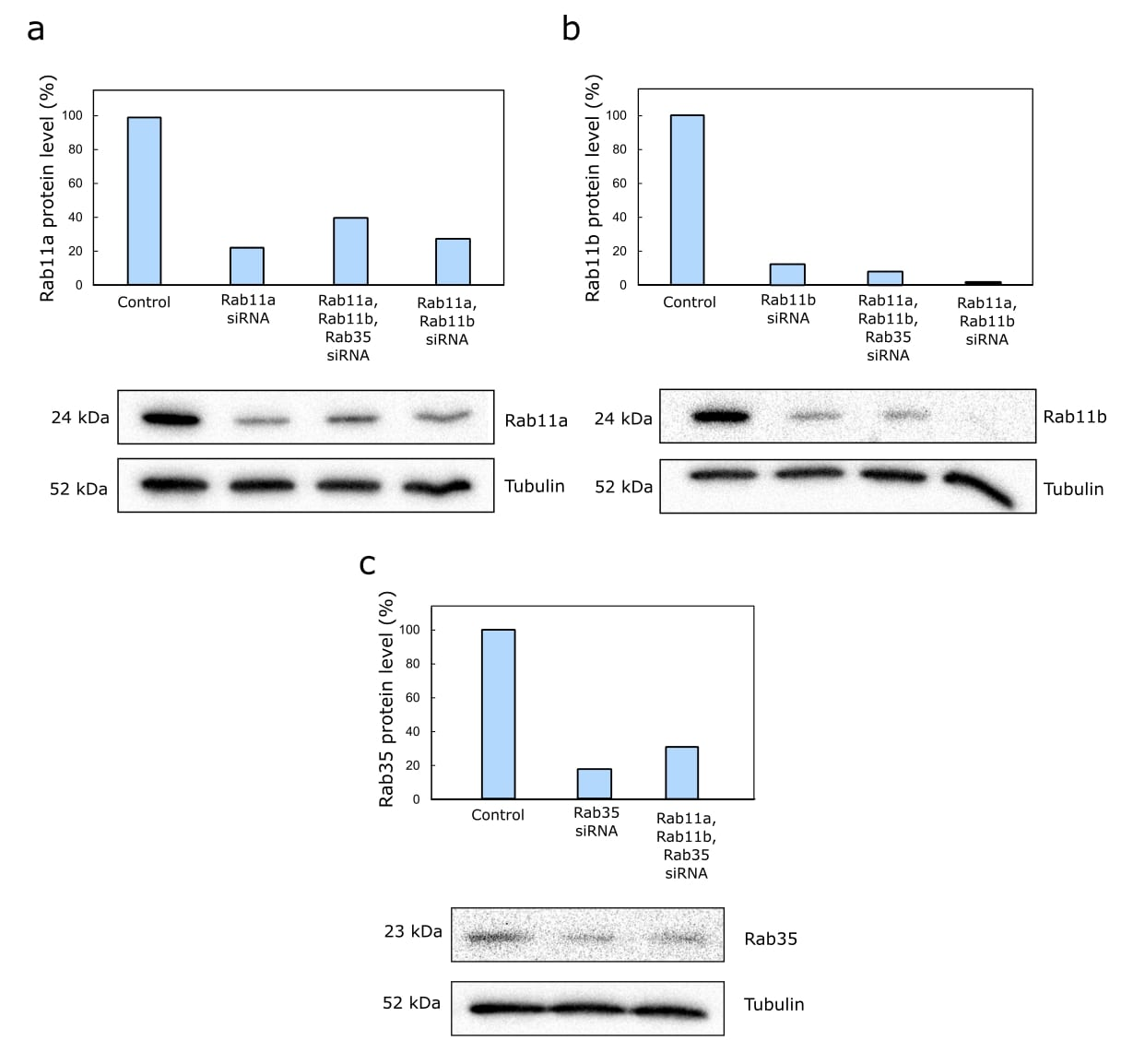

Supplement: Supplementary file 4 — Supplementary material 4: Figure 4. The expression of Rab11a, Rab11b and Rab35 remains unaffected by double or triple knockdown of Rab proteins.HeLa-wt cells transfected with individual or combined siRNAs were cultured to confluency. Cell lysates were then collected, and the relative protein levels of Rab11a, Rab11b, or Rab35 were assessed by Western blot analysis. Quantification of each is shown above the corresponding WB bands. β-tubulin served as a loading control, and protein expression levels were normalised to intracellular tubulin [file 40659_2025_638_MOESM4_ESM.tiff]

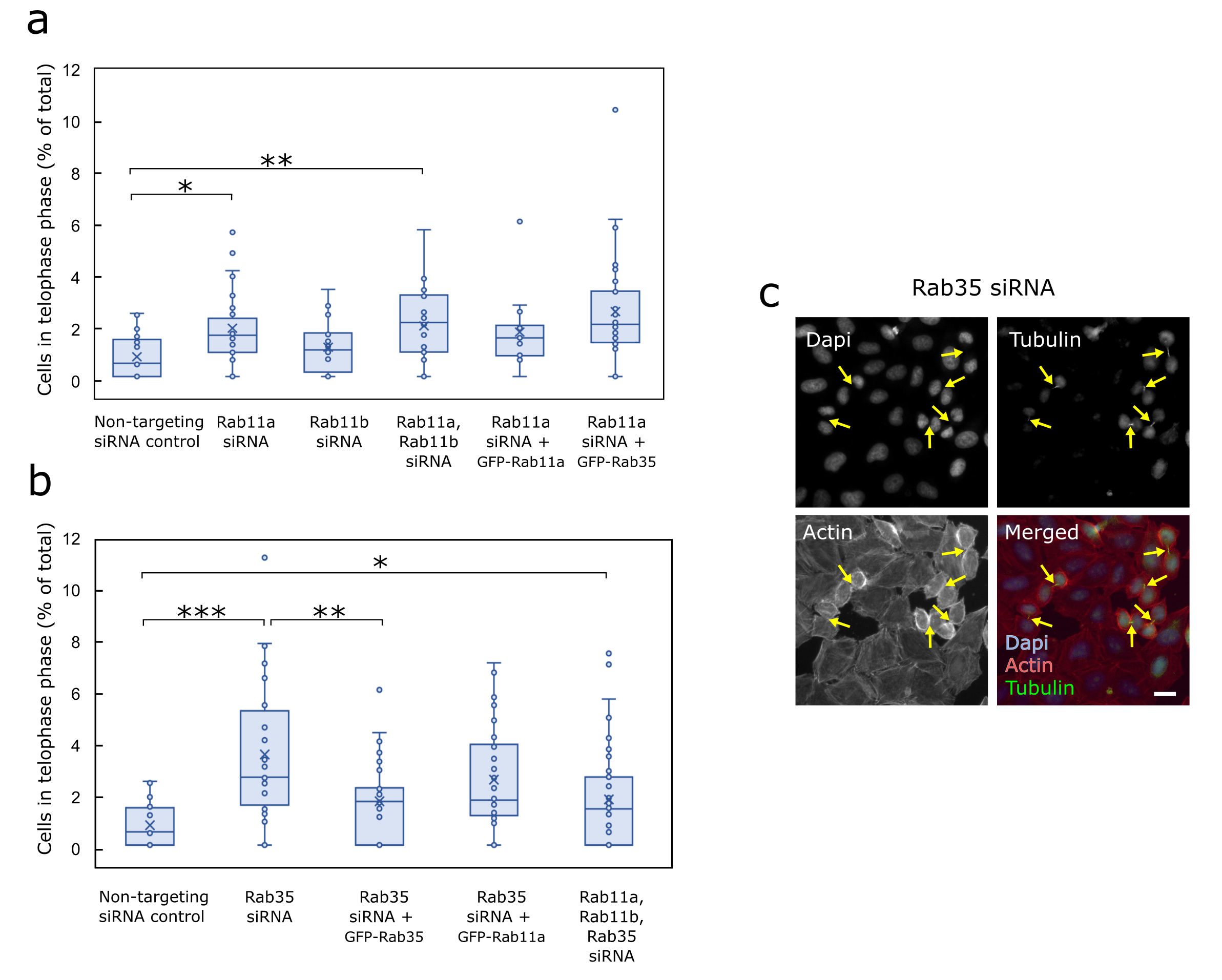

Supplement: Supplementary file 5 — Supplementary material 5: Figure 5. Restoring normal telophase progression following Rab11a, Rab11b, and Rab35 depletion.HeLa-wt cells treated with non-targeting siRNA control or specific siRNAs were subsequently transfected with siRNA-resistant Rab11a-GFP or Rab35-GFP plasmids, followed by fixation and staining with DAPI, phalloidin, and anti-acetylated-α-tubulin primary antibody. The telophase phase count was then performed manually. The image shows the rescue of the KD with the same gene or overexpression of a different gene. Asterisks (*) indicate statistically significant differences. The data shown are the means and S.D. derived from at least three independent experiments. Unless otherwise stated, at least 30 images per study group were acquired using 20× magnification.The images depict an increased number of cells being delayed or arrested in telophase following Rab35 siRNA treatment. This was identified by staining the ICB with an anti-acetylated-α-tubulin primary antibody. Arrows highlight various ICBs formed during late telophase. [file 40659_2025_638_MOESM5_ESM.tiff]

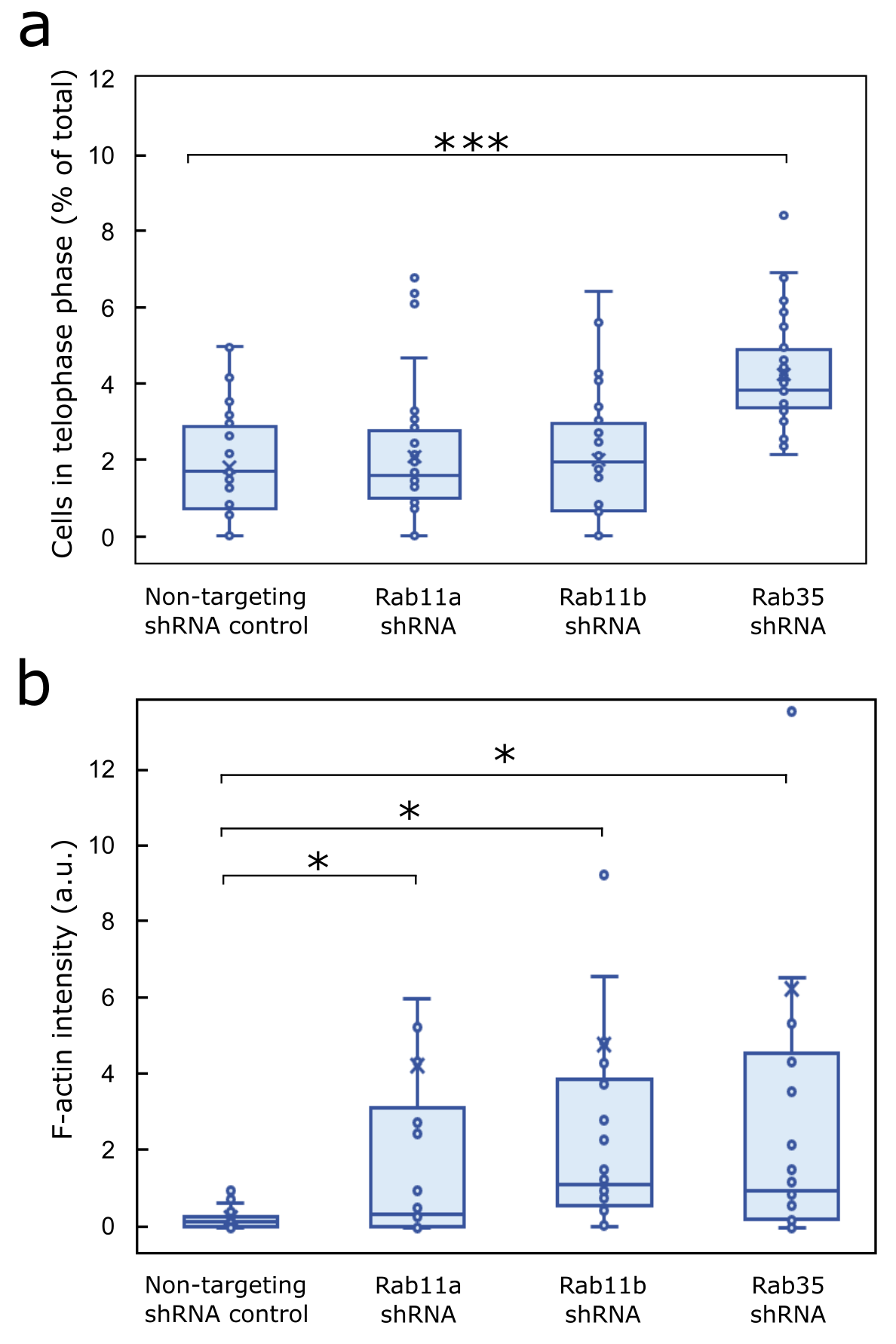

Supplement: Supplementary file 6 — Supplementary material 6: Figure 6. The downregulation of Rabs via shRNA causes telophase arrest or delay in more cells, accompanied by elevated F-actin at the intercellular bridge.Stable cell lines expressing different shRNAs were fixed and stained with DAPI, phalloidin, and an anti-acetylated-α-tubulin primary antibody. The number of cells in telophase was manually counted. Asterisks (*) indicate statistically significant differences. At least 30 images were examined for each study group using 20× magnification. A minimum of three independent experiments were conducted in total. Results are shown as mean ± standard deviation.The cells were prepared as specified in Supplemental Fig 6A. Actin enrichment at the ICB was then assessed by confocal microscopy using a 60× oil immersion objective. The actin levels at the ICB were quantified by comparing control cells to those treated with individual shRNA. Asterisks (*) indicate statistically significant differences. For each study group, at least 10 different cells were examined. A minimum of three independent experiments were conducted in total. Results are shown as mean ± standard deviation. [file 40659_2025_638_MOESM6_ESM.tiff]
